# Supplementary material for: Problem-solving skills are predicted by technical innovations in the wild and brain size in passerines
Source: Nat Ecol Evol. 2024 Feb 22;8(4):806–16. doi: 10.1038/s41559-024-02342-7 (PMC11009111; doi:10.1038/s41559-024-02342-7)
Supplement: Supplementary file 1 — Supplementary Tables 1–7. [file 41559_2024_2342_MOESM1_ESM.pdf]

nature ecology & evolution

Article

https://doi.org/10.1038/s41559-024-02342-7

Problem-solving skills are predicted by technical innovations in the wild and brain size in passerines

In the format provided by the authors and unedited

Supplementary Tables

Supplementary Table 1. Study species list, two-letter codes in figures and sample size.

Species (common)	Species (scientific)	Family	Two-letter code	*n*
American goldfinch	*Spinus tristis*	Fringillidae	AG	13
American robin	*Turdus migratorius*	Turdidae	AR	12
Black-capped chickadee	*Poecile atricapillus*	Paridae	BC	19
Blue Jay	*Cyanocitta cristata*	Corvidae	BJ	13
Brown-headed cowbird	*Molothrus ater*	Icteridae	BH	14
Chipping sparrow	*Spizella passerina*	Passerellidae	CS	12
Eastern phoebe	*Sayornis phoebe*	Tyrannidae	EP	13
European starling	*Sturnus vulgaris*	Sturnidae	ES	16
Gray catbird	*Dumetella carolinensis*	Mimidae	GC	15
House wren	*Troglodytes aedon*	Troglodytidae	HW	13
Tufted titmouse	*Baeolophus bicolor*	Paridae	TT	13
White-breasted nuthatch	*Sitta carolinensis*	Sittidae	WN	13
White-throated sparrow	*Zonotrichia albicollis*	Passerellidae	WS	12
Canary	*Serinus canaria*	Fringillidae	DC	13
Zebra finch	*Taeniopygia guttata*	Estrildidae	ZF	12
Total				203

Supplementary Table 2. Interspecific associations between all cognitive traits. Interspecific associations between all cognitive traits. Tests of associations between species means of a) performance on the four problem-solving tasks, b) each measured cognitive trait, c) cognitive and personality traits, d-g) cognitive traits and published metrics of innovation and brain size. P_{MCMC} are from simple MCMCglmm models with only both variables and phylogenetic correction, $P_{MCMC.adj}$ are adjusted P_{MCMC} using Benjamini & Hochberg multiple test correction. Bold text shows significant relationships. Sample size: 15 species (all), 14 species (songbird) or 13 species (wild).

a) Between problem-solving tasks

Dependent	Independent	post.mean	l-95% C.I.	u-95% C.I.	eff.samp	P_{MCMC}	$P_{MCMC.adj}$
**Lid-flipping**	**Lid-pulling**	**0.928**	**0.520**	**1.321**	**903**	**0.0012**	**0.0071**
**Lid-flipping**	**Lid-piercing**	**0.787**	**0.409**	**1.154**	**976**	**0.0025**	**0.0076**
**Lid-flipping**	**Stick-pulling**	**0.845**	**0.192**	**1.479**	**534**	**0.0194**	**0.0194**
**Lid-piercing**	**Lid-pulling**	**0.754**	**0.249**	**1.261**	**930**	**0.0093**	**0.0125**
**Stick-pulling**	**Lid-pulling**	**0.592**	**0.196**	**0.986**	**986**	**0.0079**	**0.0125**
**Stick-pulling**	**Lid-piercing**	**0.469**	**0.141**	**0.795**	**1005**	**0.0104**	**0.0125**

b) Between all cognitive tasks

Dependent	Independent	post.mean	l-95% C.I.	u-95% C.I.	eff.samp	P_{MCMC}	$P_{MCMC.adj}$
Problem-solving	Associative learning	0.116	-0.136	0.369	999	0.3471	0.4166
Problem-solving	Reversal learning	0.072	-0.005	0.149	998	0.0680	0.2040
Problem-solving	Self-control	-0.034	-0.234	0.166	1028	0.7203	0.7203
**Reversal learning**	**Associative learning**	**2.812**	**2.173**	**3.440**	**879**	**0.0010**	**0.0060**
Self-control	Reversal learning	-0.131	-0.387	0.133	789	0.3057	0.4166
Associative learning	Self-control	-0.236	-0.691	0.222	850	0.2906	0.4166

c) Between cognitive tasks and personality

Dependent	Independent	post.mean	l-95% C.I.	u-95% C.I.	eff.samp	P_{MCMC}	$P_{MCMC.adj}$
Problem-solving	Shyness	1.186	-2.995	5.666	863	0.5728	0.7872
Problem-solving	Neophobia	-1.461	-4.142	1.261	817	0.2405	0.7872
Associative learning	Shyness	-2.188	-11.201	6.986	1008	0.5904	0.7872
Associative learning	Neophobia	3.357	-2.450	9.156	948	0.2215	0.7872
Reversal learning	Shyness	-7.879	-32.940	17.364	999	0.4929	0.7872
Reversal learning	Neophobia	1.283	-17.185	19.435	1012	0.8664	0.9406
Self-control	Shyness	-0.608	-13.425	11.756	1044	0.9406	0.9406
Self-control	Neophobia	-3.138	-11.995	5.364	889	0.4272	0.7872

d) Problem-solving vs published metrics of innovation and brain size

Dependent	Independent	post.mean	l-95% C.I.	u-95% C.I.	eff.samp	P_{MCMC}	$P_{MCMC.adj}$
**Problem-solving**	**Technical innovation**	**-1.975**	**-3.625**	**-0.355**	**646**	**0.0212**	**0.0282**
Problem-solving	Food innovation	0.001	-1.807	1.818	823	0.9739	0.9739
**Problem-solving**	**Absolute brain size**	**-5.848**	**-9.804**	**-1.875**	**996**	**0.0075**	**0.0282**
**Problem-solving**	**Relative brain size**	**-6.217**	**-11.280**	**-1.170**	**1003**	**0.0209**	**0.0282**

e) Associative learning vs published metrics of innovation and brain size

Dependent	Independent	post.mean	l-95% C.I.	u-95% C.I.	eff.samp	P_{MCMC}	$P_{MCMC.adj}$
Associative learning	Technical innovation	-0.043	-4.300	4.273	1003	0.9708	0.9708
Associative learning	Food innovation	0.547	-3.637	4.758	590	0.7983	0.9708
Associative learning	Absolute brain size	-2.782	-15.224	9.705	988	0.6393	0.9708
Associative learning	Relative brain size	1.546	-13.235	16.397	934	0.8220	0.9708

f) Reversal learning vs published metrics of innovation and brain size

Dependent	Independent	post.mean	l-95% C.I.	u-95% C.I.	eff.samp	P_{MCMC}	$P_{MCMC.adj}$
Reversal learning	Technical innovation	-1.066	-13.500	11.307	1022	0.8642	0.8642
Reversal learning	Food innovation	1.152	-10.530	13.014	914	0.8408	0.8642
Reversal learning	Absolute brain size	-23.413	-56.691	9.937	1003	0.1561	0.6246
Reversal learning	Relative brain size	-13.238	-54.959	28.339	1019	0.5059	0.8642

g) Self-control vs published metrics of innovation and brain size

Dependent	Independent	post.mean	l-95% C.I.	u-95% C.I.	eff.samp	P_{MCMC}	$P_{MCMC.adj}$
Self-control	Technical innovation	1.070	-4.491	6.664	963	0.6921	0.6921
Self-control	Food innovation	1.329	-4.132	6.805	420	0.6312	0.6921
Self-control	Absolute brain size	7.953	-7.442	23.367	980	0.2924	0.6921
Self-control	Relative brain size	-5.119	-23.861	13.494	1019	0.5677	0.6921

Supplementary Table 3. Models assessing interindividual associations between each cognitive task. Models include each combination of measured traits as explanatory or response variables (trials to succeed), and species as a random effect. P_{LMM} are computed from linear mixed models, P_{MCMC} from equivalent MCMCglmm with phylogenetic correction, $P_{LMM.adj}$ and $P_{MCMC.adj}$ are adjusted p-values following Benjamini & Hochberg multiple test correction. Bold text shows significant relationship. Sample size: 203 individuals (15 species), 190 individuals (14 songbird species), or 178 individuals (13 wild species).

Dependent	Independent	Estimate	Std. Error	df	*t*	P_{LMM}	$P_{LMM.adj}$	P_{MCMC}	$P_{MCMC.adj}$
Learning	(Intercept)	6.705	0.699	21.400	9.596	0.0000	-	0.0225	-
	Problem-solving	0.000	0.017	193.700	0.007	0.9940	0.9940	0.9758	0.9758
Reversal learning	(Intercept)	6.896	0.728	22.444	9.477	0.0000	-	0.0212	-
	Problem-solving	-0.005	0.010	200.679	-0.534	0.5940	0.7128	0.6377	0.7652
Self-control	(Intercept)	7.077	0.704	21.800	10.053	0.0000	-	0.0206	-
	Problem-solving	-0.024	0.021	200.217	-1.142	0.2550	0.6180	0.2434	0.4911
**Learning**	(Intercept)	22.755	3.908	24.944	5.823	<0.0001	-	0.0278	-
	**Reversal learning**	**0.743**	**0.111**	**196.614**	**6.668**	**<0.0001**	**<0.0001**	**0.0010**	**0.0060**
Reversal learning	(Intercept)	16.371	2.175	25.554	7.528	0.0000	-	0.0257	-
	Self-control	-0.029	0.032	200.733	-0.903	0.3680	0.6180	0.2455	0.4911
Learning	(Intercept)	14.460	2.100	23.710	6.886	0.0000	-	0.0361	-
	Self-control	0.046	0.055	195.367	0.822	0.4120	0.6180	0.5140	0.7652

Supplementary Table 4. Interindividual associations between all cognitive traits, tested separately for each species. Estimate, R-squared (R^2), F-statistic and P_{LM} are computed with separate linear models for each species, $P_{LM.adj}$ are adjusted using Benjamini & Hochberg multiple test correction. Bold text shows significant relationships. Sample size: 203 individuals, 15 species.

a) Associative learning vs Problem-solving

Species	Estimate	R^2	F	P_{LM}	$P_{LM.adj}$
American goldfinch	0.338	-0.041	0.528	0.4825	0.9877
American robin	0.768	-0.005	0.944	0.3543	0.9877
Black-capped chickadee	-0.565	-0.031	0.465	0.5047	0.9877
Blue Jay	-1.338	-0.022	0.744	0.4067	0.9877
Brown-headed cowbird	0.344	-0.041	0.488	0.4983	0.9877
Canary	-0.518	-0.071	0.206	0.6585	0.9877
Chipping sparrow	-3.281	0.101	2.234	0.1658	0.9877
Eastern phoebe	0.417	-0.091	0.003	0.9596	0.9920
European starling	-0.019	-0.071	0.000	0.9920	0.9920
Gray catbird	-0.292	-0.072	0.062	0.8070	0.9920
House wren	-0.520	-0.086	0.047	0.8317	0.9920
Tufted titmouse	-0.334	-0.066	0.254	0.6242	0.9877
White-breasted nuthatch	0.856	-0.057	0.356	0.5630	0.9877
White-throated sparrow	-0.646	-0.053	0.444	0.5202	0.9877
Zebra finch	-0.090	-0.098	0.018	0.8965	0.9920

b) Reversal learning vs Problem-solving

Species	Estimate	R^2	F	P_{LM}	$P_{LM.adj}$
American goldfinch	-0.728	0.043	1.539	0.2406	0.6558
American robin	1.281	-0.038	0.595	0.4582	0.6872
Black-capped chickadee	-0.514	-0.052	0.116	0.7373	0.8914
Blue Jay	-2.930	-0.018	0.789	0.3935	0.6558
Brown-headed cowbird	0.632	-0.013	0.837	0.3782	0.6558
Canary	-0.345	-0.085	0.057	0.8154	0.8914
Chipping sparrow	-3.768	-0.075	0.237	0.6371	0.8687
Eastern phoebe	-18.750	0.202	4.044	0.0695	0.6558
European starling	-2.064	0.004	1.064	0.3198	0.6558
Gray catbird	-2.043	0.007	1.099	0.3137	0.6558
House wren	-4.213	0.096	2.269	0.1602	0.6558
Tufted titmouse	1.306	0.032	1.392	0.2630	0.6558
White-breasted nuthatch	0.560	-0.088	0.033	0.8592	0.8914
White-throated sparrow	0.230	-0.098	0.020	0.8914	0.8914
Zebra finch	-2.097	0.077	1.920	0.1959	0.6558

c) Self-control vs Problem-solving

Species	Estimate	R^2	F	P_{LM}	$P_{LM.adj}$
American goldfinch	0.773	0.228	4.540	0.0565	0.4092
American robin	-0.457	-0.089	0.103	0.7543	0.8704
Black-capped chickadee	-0.708	-0.027	0.526	0.4783	0.7244
Blue Jay	1.236	-0.041	0.527	0.4830	0.7244
Brown-headed cowbird	-0.523	-0.029	0.632	0.4420	0.7244
Canary	-0.699	0.096	2.275	0.1596	0.5022
Chipping sparrow	-2.769	0.254	4.746	0.0544	0.4092
Eastern phoebe	10.167	0.182	3.667	0.0818	0.4092
European starling	-1.637	0.058	1.921	0.1874	0.5022
Gray catbird	-2.279	0.037	1.537	0.2370	0.5079
House wren	-0.209	-0.086	0.050	0.8269	0.8780
Tufted titmouse	0.224	-0.071	0.208	0.6574	0.8262
White-breasted nuthatch	0.209	-0.071	0.203	0.6610	0.8262
White-throated sparrow	-0.358	0.074	1.875	0.2009	0.5022
Zebra finch	-0.058	-0.097	0.025	0.8780	0.8780

d) Associative learning vs Reversal learning

Species	Estimate	R^2	F	P_{LM}	$P_{LM.adj}$
American goldfinch	-0.155	-0.046	0.475	0.5048	0.5411
American robin	0.183	0.057	1.667	0.2257	0.4810
Black-capped chickadee	-0.012	-0.058	0.008	0.9283	0.9283
**Blue Jay**	**0.397**	**0.691**	**27.862**	**0.0003**	**0.0020**
Brown-headed cowbird	0.214	0.016	1.214	0.2921	0.4810
Canary	0.255	0.021	1.261	0.2853	0.4810
Chipping sparrow	0.091	-0.004	0.954	0.3518	0.4810
Eastern phoebe	0.407	0.240	4.798	0.0509	0.1528
**European starling**	**0.738**	**0.631**	**26.646**	**0.0001**	**0.0020**
**Gray catbird**	**0.308**	**0.229**	**5.169**	**0.0406**	0.1528
House wren	0.205	-0.015	0.819	0.3848	0.4810
Tufted titmouse	0.116	-0.046	0.475	0.5050	0.5411
**White-breasted nuthatch**	**0.268**	**0.260**	**5.224**	**0.0431**	0.1528
White-throated sparrow	-0.170	-0.013	0.858	0.3762	0.4810
Zebra finch	0.176	0.101	2.242	0.1652	0.4130

e) Reversal learning vs Self-control

Species	Estimate	R^2	F	P_{LM}	$P_{LM.adj}$
American goldfinch	-0.388	-0.013	0.845	0.3777	0.7493
American robin	0.090	-0.094	0.057	0.8157	0.9137
Black-capped chickadee	0.112	-0.053	0.091	0.7661	0.9137
**Blue Jay**	**-1.248**	**0.351**	**7.504**	**0.0193**	0.1639
Brown-headed cowbird	0.035	-0.082	0.013	0.9101	0.9137
Canary	-0.474	-0.061	0.315	0.5861	0.8791
Chipping sparrow	1.394	-0.017	0.815	0.3878	0.7493
**Eastern phoebe**	**-1.115**	**0.338**	**7.120**	**0.0219**	0.1639
European starling	0.393	-0.011	0.844	0.3739	0.7493
Gray catbird	-0.032	-0.076	0.012	0.9137	0.9137
House wren	0.700	-0.042	0.521	0.4853	0.8089
Tufted titmouse	-0.605	-0.020	0.768	0.3996	0.7493
White-breasted nuthatch	2.040	0.013	1.161	0.3043	0.7493
White-throated sparrow	-1.768	0.004	1.040	0.3318	0.7493
Zebra finch	-0.176	-0.098	0.015	0.9034	0.9137

f) Associative learning vs Self-control

Species	Estimate	R^2	F	P_{LM}	$P_{LM.adj}$
American goldfinch	0.545	0.175	3.540	0.0866	0.2190
American robin	0.014	-0.099	0.006	0.9399	0.9926
Black-capped chickadee	0.240	0.026	1.480	0.2404	0.4508
**Blue Jay**	**-0.687**	**0.518**	**13.879**	**0.0034**	0.0503
Brown-headed cowbird	-0.085	-0.069	0.159	0.6972	0.8715
Canary	0.006	-0.091	0.000	0.9926	0.9926
Chipping sparrow	0.876	0.241	4.492	0.0601	0.2190
Eastern phoebe	-0.401	0.011	1.131	0.3104	0.5174
European starling	0.248	-0.042	0.393	0.5407	0.8094
Gray catbird	0.275	0.147	3.412	0.0876	0.2190
**House wren**	**1.779**	**0.435**	**10.232**	**0.0085**	0.0636
Tufted titmouse	-0.499	0.058	1.745	0.2133	0.4508
White-breasted nuthatch	0.192	-0.087	0.042	0.8413	0.9707
White-throated sparrow	0.598	-0.068	0.304	0.5936	0.8094
Zebra finch	-1.027	0.244	4.546	0.0588	0.2190

Supplementary Table 5. Full MCMCglmm phylogenetic models assessing the relationships between all measured behaviours and published metrics. Full models (a) are the initial models with all potential covariates and final models (b) are the models that contain only the significant effects (if any) after stepwise variable selection. Significant effects are highlighted in bold and random effects are in italics. Innovation variables are corrected for research effort. Sample size: 203 individuals, 15 Species.

Model	Dependent	Independent	post.mean	l-95% C.I.	u-95% C.I.	eff.samp	P_{MCMC}
1. a) Full	**Shyness**	(Intercept)	-1.179	-4.510	2.124	1011	0.490
		Technical innovation	-0.210	-0.436	0.019	997	0.074
		Body condition	-0.106	-0.636	0.433	1010	0.686
		**Captive status: wild** (vs dom)	**0.601**	**0.146**	**1.045**	**1017**	**0.016**
		Dietary generalism	0.109	-0.027	0.250	994	0.111
		Reward: seeds (vs worms)	-0.011	-0.233	0.209	1021	0.927
		Fasting time	1.606	-1.380	4.600	1011	0.302
		Neophobia	-0.094	-0.236	0.051	1023	0.204
		Phylogeny	*0.030*	*0.000*	*0.116*	*802*	
		Capture site	*0.005*	*0.000*	*0.016*	*994*	
		Species	*0.027*	*0.000*	*0.076*	*991*	
1. b) Final	**Shyness**	(Intercept)	0.801	0.370	1.231	1011	0.003
		**Captive status: wild** (vs dom)	**0.580**	**0.188**	**0.971**	**1009**	**0.007**
		Phylogeny	*0.037*	*0.000*	*0.127*	*796*	
		Capture site	*0.005*	*0.000*	*0.018*	*977*	
		Species	*0.030*	*0.000*	*0.080*	*906*	
2. a) Full	**Shyness**	(Intercept)	-0.331	-3.587	2.923	1007	0.844
		Food innovation	-0.042	-0.284	0.202	1015	0.720
		Body condition	-0.211	-0.802	0.380	1012	0.474
		**Captive status: wild** (vs dom)	**0.568**	**0.070**	**1.072**	**1012**	**0.034**
		Dietary generalism	0.093	-0.082	0.271	1022	0.275
		Reward: seeds (vs worms)	0.018	-0.218	0.255	1006	0.884
		Fasting time	1.072	-1.882	4.013	1027	0.479
		Neophobia	-0.099	-0.247	0.048	1001	0.190
		Phylogeny	*0.053*	*0.000*	*0.192*	*736*	
		Capture site	*0.005*	*0.000*	*0.017*	*981*	
		Species	*0.039*	*0.000*	*0.109*	*919*	
2. b) Final	**Shyness**	(Intercept)	0.801	0.370	1.231	1011	0.003
		**Captive status: wild** (vs dom)	**0.580**	**0.188**	**0.971**	**1009**	**0.007**
		Phylogeny	*0.037*	*0.000*	*0.127*	*796*	
		Capture site	*0.005*	*0.000*	*0.018*	*977*	
		Species	*0.030*	*0.000*	*0.080*	*906*	
3. a) Full	**Shyness**	(Intercept)	0.752	-2.975	4.506	1013	0.694
		Absolute brain size	0.771	-0.696	2.251	970	0.295
		Body condition	-0.666	-1.667	0.338	986	0.189
		**Captive status: wild** (vs dom)	**0.530**	**0.040**	**1.015**	**1010**	**0.042**
		Dietary generalism	0.062	-0.093	0.217	996	0.407
		Reward: seeds (vs worms)	0.049	-0.181	0.274	1015	0.675
		Fasting time	0.808	-2.165	3.793	1005	0.595

		Neophobia	-0.087	-0.234	0.058	1009	0.248
		Phylogeny	*0.056*	*0.000*	*0.179*	*782*	
		Capture site	*0.005*	*0.000*	*0.017*	*958*	
		Species	*0.030*	*0.000*	*0.092*	*865*	
3. b) Final	**Shyness**	(Intercept)	0.801	0.370	1.231	1011	0.003
		**Captive status: wild** (vs dom)	**0.580**	**0.188**	**0.971**	**1009**	**0.007**
		Phylogeny	*0.037*	*0.000*	*0.127*	*796*	
		Capture site	*0.005*	*0.000*	*0.018*	*977*	
		Species	*0.030*	*0.000*	*0.080*	*906*	
4. a) Full	**Shyness**	(Intercept)	0.128	-3.154	3.410	1006	0.939
		Relative brain size	0.427	-0.420	1.327	994	0.308
		Body condition	-0.293	-0.881	0.285	997	0.310
		**Captive status: wild** (vs dom)	**0.527**	**0.025**	**1.009**	**1012**	**0.043**
		Dietary generalism	0.067	-0.087	0.224	1029	0.372
		Reward: seeds (vs worms)	0.048	-0.181	0.272	1004	0.680
		Fasting time	0.838	-2.099	3.791	1011	0.581
		Neophobia	-0.097	-0.240	0.050	1006	0.193
		Phylogeny	*0.059*	*0.000*	*0.192*	*790*	
		Capture site	*0.005*	*0.000*	*0.017*	*978*	
		Species	*0.031*	*0.000*	*0.095*	*872*	
4. b) Final	**Shyness**	(Intercept)	0.801	0.370	1.231	1011	0.003
		**Captive status: wild** (vs dom)	**0.580**	**0.188**	**0.971**	**1009**	**0.007**
		Phylogeny	*0.037*	*0.000*	*0.127*	*796*	
		Capture site	*0.005*	*0.000*	*0.018*	*977*	
		Species	*0.030*	*0.000*	*0.080*	*906*	
5. a) Full	**Neophobia**	(Intercept)	-2.229	-5.484	1.030	1004	0.183
		Technical innovation	-0.139	-0.512	0.215	987	0.429
		Body condition	0.466	-0.298	1.290	942	0.236
		**Captive status: wild** (vs dom)	**-0.671**	**-1.303**	**-0.045**	**963**	**0.038**
		Dietary generalism	0.126	-0.098	0.350	984	0.241
		Reward: seeds (vs worms)	-0.171	-0.390	0.051	1010	0.134
		Fasting time	2.210	-0.610	5.027	1034	0.128
		Shyness	-0.099	-0.232	0.035	1008	0.153
		Phylogeny	*0.120*	*0.000*	*0.437*	*592*	
		Capture site	*0.005*	*0.000*	*0.017*	*974*	
		Species	*0.075*	*0.000*	*0.211*	*755*	
5. b) Final	**Neophobia**	(Intercept)	-3.417	-6.310	-0.569	1002	0.023
		**Fasting time**	**3.360**	**0.983**	**5.770**	**999**	**0.008**
		Phylogeny	*0.036*	*0.000*	*0.155*	*741*	
		Capture site	*0.006*	*0.000*	*0.023*	*962*	
		Species	*0.081*	*0.002*	*0.174*	*985*	
6. a) Full	**Neophobia**	(Intercept)	-2.414	-5.496	0.679	1021	0.129
		**Food innovation**	**-0.287**	**-0.557**	**-0.026**	**992**	**0.034**
		Body condition	0.355	-0.260	0.996	1003	0.257
		**Captive status: wild** (vs dom)	**-0.618**	**-1.135**	**-0.088**	**1007**	**0.025**
		Dietary generalism	0.196	0.002	0.395	947	0.052

		Reward: seeds (vs worms)	-0.221	-0.442	0.002	1012	0.054
		Fasting time	2.365	-0.403	5.189	1020	0.101
		Shyness	-0.092	-0.225	0.040	1016	0.180
		Phylogeny	*0.078*	*0.000*	*0.255*	*718*	
		Capture site	*0.005*	*0.000*	*0.017*	*977*	
		Species	*0.042*	*0.000*	*0.128*	*810*	
6. b) Final	**Neophobia**	(Intercept)	-3.217	-6.022	-0.404	1002	0.030
		**Food innovation**	**-0.267**	**-0.509**	**-0.030**	**976**	**0.030**
		**Captive status: wild** (vs dom)	**-0.602**	**-1.064**	**-0.148**	**993**	**0.015**
		**Dietary generalism**	**0.199**	**0.028**	**0.376**	**964**	**0.027**
		**Reward: seeds** (vs worms)	**-0.233**	**-0.453**	**-0.011**	**1005**	**0.041**
		**Fasting time**	**3.338**	**1.005**	**5.608**	**1009**	**0.007**
		Phylogeny	*0.055*	*0.000*	*0.181*	*745*	
		Capture site	*0.005*	*0.000*	*0.017*	*971*	
		Species	*0.032*	*0.000*	*0.095*	*825*	
7. a) Full	Neophobia	(Intercept)	-3.213	-6.920	0.430	1002	0.090
		Absolute brain size	-1.201	-2.998	0.576	997	0.182
		Body condition	0.965	-0.183	2.130	1026	0.097
		Captive status: wild (vs dom)	-0.571	-1.224	0.084	986	0.087
		Dietary generalism	0.130	-0.087	0.349	1003	0.215
		Reward: seeds (vs worms)	-0.163	-0.377	0.054	1009	0.143
		Fasting time	2.299	-0.520	5.104	1007	0.114
		Shyness	-0.089	-0.221	0.043	1017	0.189
		Phylogeny	*0.076*	*0.000*	*0.326*	*607*	
		Capture site	*0.005*	*0.000*	*0.018*	*981*	
		Species	*0.091*	*0.000*	*0.227*	*871*	
7. b) Final	**Neophobia**	(Intercept)	-3.417	-6.310	-0.569	1002	0.023
		**Fasting time**	**3.360**	**0.983**	**5.770**	**999**	**0.008**
		Phylogeny	*0.036*	*0.000*	*0.155*	*741*	
		Capture site	*0.006*	*0.000*	*0.023*	*962*	
		Species	*0.081*	*0.002*	*0.174*	*985*	
8. a) Full	**Neophobia**	(Intercept)	-1.826	-5.024	1.410	1020	0.271
		Relative brain size	0.154	-1.057	1.410	940	0.790
		Body condition	0.356	-0.364	1.124	991	0.341
		**Captive status: wild** (vs dom)	**-0.691**	**-1.318**	**-0.065**	**1004**	**0.035**
		Dietary generalism	0.099	-0.117	0.313	998	0.334
		Reward: seeds (vs worms)	-0.153	-0.373	0.067	1008	0.175
		Fasting time	2.057	-0.764	4.849	1045	0.156
		Shyness	-0.095	-0.228	0.038	1023	0.165
		Phylogeny	*0.125*	*0.000*	*0.426*	*650*	
		Capture site	*0.005*	*0.000*	*0.016*	*965*	
		Species	*0.070*	*0.000*	*0.210*	*741*	
8. b) Final	**Neophobia**	(Intercept)	-3.417	-6.310	-0.569	1002	0.023
		**Fasting time**	**3.360**	**0.983**	**5.770**	**999**	**0.008**
		Phylogeny	*0.036*	*0.000*	*0.155*	*741*	
		Capture site	*0.006*	*0.000*	*0.023*	*962*	

		Species	*0.081*	*0.002*	*0.174*	*985*	
9. a) Full	**Problem-solving**	(Intercept)	-0.102	-1.988	1.751	1005	0.921
		Technical innovation	-0.162	-0.373	0.052	969	0.129
		Body condition	-0.169	-0.570	0.237	992	0.400
		Captive status: wild (vs dom)	0.056	-0.314	0.432	1012	0.761
		Dietary generalism	-0.051	-0.174	0.073	1013	0.384
		Reward: seeds (vs worms)	0.051	-0.075	0.175	1025	0.428
		Fasting time	0.768	-0.839	2.359	1004	0.351
		Neophobia	0.059	-0.019	0.137	1002	0.139
		**Shyness**	**0.110**	**0.036**	**0.185**	**1012**	**0.005**
		Phylogeny	*0.041*	*0.000*	*0.119*	*812*	
		Capture site	*0.006*	*0.000*	*0.022*	*970*	
		Species	*0.020*	*0.000*	*0.061*	*866*	
9. b) Final	**Problem-solving**	(Intercept)	0.565	0.319	0.831	991	0.001
		**Technical innovation**	**-0.204**	**-0.360**	**-0.047**	**970**	**0.015**
		**Shyness**	**0.100**	**0.027**	**0.173**	**1013**	**0.008**
		Phylogeny	*0.034*	*0.000*	*0.094*	*851*	
		Capture site	*0.004*	*0.000*	*0.015*	*966*	
		Species	*0.016*	*0.000*	*0.045*	*866*	
10. a) Full	**Problem-solving**	(Intercept)	0.351	-1.488	2.185	993	0.706
		Food innovation	0.067	-0.132	0.268	1010	0.479
		Body condition	-0.290	-0.675	0.104	1009	0.147
		Captive status: wild (vs dom)	0.045	-0.328	0.423	1009	0.798
		Dietary generalism	-0.102	-0.240	0.036	995	0.133
		Reward: seeds (vs worms)	0.073	-0.053	0.200	1015	0.270
		Fasting time	0.623	-0.975	2.209	999	0.447
		Neophobia	0.064	-0.013	0.144	1003	0.110
		**Shyness**	**0.113**	**0.039**	**0.188**	**1016**	**0.004**
		Phylogeny	*0.067*	*0.001*	*0.158*	*944*	
		Capture site	*0.006*	*0.000*	*0.021*	*976*	
		Species	*0.013*	*0.000*	*0.053*	*810*	
10. b) Final	**Problem-solving**	(Intercept)	0.572	0.241	0.911	1012	0.003
		**Shyness**	**0.103**	**0.031**	**0.177**	**1025**	**0.007**
		Phylogeny	*0.074*	*0.001*	*0.160*	*917*	
		Capture site	*0.005*	*0.000*	*0.016*	*976*	
		Species	*0.014*	*0.000*	*0.052*	*788*	
11. a) Full	**Problem-solving**	(Intercept)	-0.894	-2.982	1.213	1014	0.408
		**Absolute brain size**	**-1.007**	**-1.929**	**-0.074**	**1013**	**0.041**
		Body condition	0.249	-0.364	0.851	1014	0.420
		Captive status: wild (vs dom)	0.098	-0.252	0.454	1000	0.568
		Dietary generalism	-0.052	-0.162	0.056	996	0.327
		Reward: seeds (vs worms)	0.059	-0.064	0.182	1012	0.352
		Fasting time	0.857	-0.751	2.442	1007	0.300
		Neophobia	0.055	-0.022	0.132	1004	0.169
		**Shyness**	**0.122**	**0.047**	**0.196**	**1006**	**0.002**
		Phylogeny	*0.042*	*0.000*	*0.106*	*924*	

		Capture site	*0.008*	*0.000*	*0.025*	*974*	
		Species	*0.012*	*0.000*	*0.041*	*847*	
11. b) Final	**Problem-solving**	(Intercept)	0.539	0.267	0.822	1010	0.002
		**Absolute brain size**	**-0.588**	**-1.014**	**-0.160**	**1005**	**0.011**
		**Shyness**	**0.108**	**0.036**	**0.181**	**1017**	**0.004**
		Phylogeny	*0.047*	*0.001*	*0.103*	*953*	
		Capture site	*0.005*	*0.000*	*0.016*	*971*	
		Species	*0.008*	*0.000*	*0.031*	*851*	
12. a) Full	**Problem-solving**	(Intercept)	-0.063	-1.878	1.770	1014	0.943
		Relative brain size	-0.559	-1.192	0.069	998	0.078
		Body condition	-0.201	-0.575	0.182	1002	0.289
		Captive status: wild (vs dom)	0.090	-0.270	0.452	1011	0.603
		Dietary generalism	-0.059	-0.172	0.056	1009	0.287
		Reward: seeds (vs worms)	0.055	-0.070	0.179	998	0.385
		Fasting time	0.776	-0.819	2.360	1011	0.343
		Neophobia	0.064	-0.014	0.142	1001	0.111
		**Shyness**	**0.120**	**0.045**	**0.195**	**1018**	**0.002**
		Phylogeny	*0.047*	*0.000*	*0.119*	*900*	
		Capture site	*0.006*	*0.000*	*0.022*	*976*	
		Species	*0.014*	*0.000*	*0.049*	*846*	
12. b) Final	**Problem-solving**	(Intercept)	0.590	0.319	0.873	1006	0.001
		**Relative brain size**	**-0.673**	**-1.195**	**-0.159**	**1025**	**0.015**
		**Shyness**	**0.113**	**0.040**	**0.186**	**1016**	**0.003**
		Phylogeny	*0.046*	*0.000*	*0.107*	*911*	
		Capture site	*0.005*	*0.000*	*0.015*	*980*	
		Species	*0.011*	*0.000*	*0.038*	*822*	
13. a) Full	**Associative learning**	(Intercept)	0.338	-1.273	1.945	1029	0.684
		Technical innovation	0.008	-0.116	0.139	1030	0.917
		Body condition	-0.227	-0.516	0.055	1000	0.109
		Captive status: wild (vs dom)	0.071	-0.172	0.318	1006	0.557
		Dietary generalism	-0.022	-0.098	0.053	1008	0.542
		Reward: seeds (vs worms)	-0.024	-0.139	0.093	1000	0.689
		Fasting time	0.993	-0.449	2.443	1025	0.183
		**Neophobia**	**0.086**	**0.015**	**0.156**	**1007**	**0.019**
		Shyness	-0.009	-0.079	0.059	1002	0.791
		Phylogeny	*0.016*	*0.000*	*0.043*	*965*	
		Capture site	*0.002*	*0.000*	*0.007*	*986*	
		Species	*0.006*	*0.000*	*0.018*	*964*	
13. b) Final	**Associative learning**	(Intercept)	1.198	1.054	1.347	999	0.001
		**Neophobia**	**0.082**	**0.018**	**0.147**	**1004**	**0.014**
		Phylogeny	*0.012*	*0.000*	*0.030*	*989*	
		Capture site	*0.002*	*0.000*	*0.006*	*978*	
		Species	*0.004*	*0.000*	*0.012*	*986*	
14. a) Full	**Associative learning**	(Intercept)	0.516	-1.063	2.084	1019	0.521
		Food innovation	0.080	-0.026	0.185	1011	0.135
		Body condition	-0.235	-0.494	0.020	1000	0.068

		Captive status: wild (vs dom)	0.071	-0.164	0.301	1018	0.531
		Dietary generalism	-0.047	-0.120	0.027	1008	0.197
		Reward: seeds (vs worms)	0.000	-0.112	0.114	1023	0.977
		Fasting time	0.877	-0.566	2.322	1012	0.237
		**Neophobia**	**0.098**	**0.027**	**0.170**	**1020**	**0.007**
		Shyness	-0.009	-0.076	0.059	1017	0.803
		Phylogeny	*0.009*	*0.000*	*0.029*	*952*	
		Capture site	*0.002*	*0.000*	*0.007*	*968*	
		Species	*0.005*	*0.000*	*0.016*	*991*	
14. b) Final	**Associative learning**	(Intercept)	1.198	1.054	1.347	999	0.001
		**Neophobia**	**0.082**	**0.018**	**0.147**	**1004**	**0.014**
		Phylogeny	*0.012*	*0.000*	*0.030*	*989*	
		Capture site	*0.002*	*0.000*	*0.006*	*978*	
		Species	*0.004*	*0.000*	*0.012*	*986*	
15. a) Full	**Associative learning**	(Intercept)	0.494	-1.326	2.308	1020	0.596
		Absolute brain size	0.141	-0.556	0.867	1011	0.711
		Body condition	-0.299	-0.795	0.178	1017	0.219
		Captive status: wild (vs dom)	0.063	-0.183	0.309	1016	0.605
		Dietary generalism	-0.024	-0.100	0.049	1007	0.500
		Reward: seeds (vs worms)	-0.023	-0.136	0.090	1023	0.692
		Fasting time	0.965	-0.486	2.416	1008	0.200
		**Neophobia**	**0.086**	**0.016**	**0.157**	**1026**	**0.018**
		Shyness	-0.011	-0.079	0.057	1001	0.758
		Phylogeny	*0.015*	*0.000*	*0.042*	*982*	
		Capture site	*0.002*	*0.000*	*0.007*	*985*	
		Species	*0.006*	*0.000*	*0.018*	*976*	
15. b) Final	**Associative learning**	(Intercept)	1.198	1.054	1.347	999	0.001
		**Neophobia**	**0.082**	**0.018**	**0.147**	**1004**	**0.014**
		Phylogeny	*0.012*	*0.000*	*0.030*	*989*	
		Capture site	*0.002*	*0.000*	*0.006*	*978*	
		Species	*0.004*	*0.000*	*0.012*	*986*	
16. a) Full	**Associative learning**	(Intercept)	0.318	-1.282	1.926	1011	0.702
		Relative brain size	0.000	-0.410	0.408	1001	0.966
		Body condition	-0.221	-0.503	0.057	1019	0.114
		Captive status: wild (vs dom)	0.070	-0.177	0.319	991	0.569
		Dietary generalism	-0.021	-0.095	0.053	1018	0.549
		Reward: seeds (vs worms)	-0.026	-0.140	0.088	1010	0.661
		Fasting time	1.003	-0.448	2.449	1010	0.180
		**Neophobia**	**0.086**	**0.015**	**0.156**	**1009**	**0.019**
		Shyness	-0.009	-0.078	0.059	1014	0.786
		Phylogeny	*0.016*	*0.000*	*0.044*	*970*	
		Capture site	*0.002*	*0.000*	*0.007*	*958*	
		Species	*0.006*	*0.000*	*0.018*	*969*	
16. b) Final	**Associative learning**	(Intercept)	1.198	1.054	1.347	999	0.001
		**Neophobia**	**0.082**	**0.018**	**0.147**	**1004**	**0.014**
		Phylogeny	*0.012*	*0.000*	*0.030*	*989*	

		Capture site	*0.002*	*0.000*	*0.006*	*978*	
		Species	*0.004*	*0.000*	*0.012*	*986*	
17. a) Full	**Reversal learning**	(Intercept)	1.971	0.481	3.445	1018	0.010
		Technical innovation	0.002	-0.162	0.163	1000	0.969
		Body condition	-0.123	-0.442	0.198	996	0.440
		Captive status: wild (vs dom)	-0.033	-0.347	0.275	1001	0.818
		Dietary generalism	-0.037	-0.134	0.062	1010	0.431
		**Reward: seeds (vs worms)**	**-0.142**	**-0.244**	**-0.040**	**1026**	**0.007**
		Fasting time	0.025	-1.252	1.308	1026	0.965
		Neophobia	0.008	-0.055	0.071	1006	0.795
		**Shyness**	**-0.091**	**-0.150**	**-0.032**	**1005**	**0.003**
		Phylogeny	*0.032*	*0.000*	*0.082*	*935*	
		Capture site	*0.005*	*0.000*	*0.015*	*979*	
		Species	*0.011*	*0.000*	*0.035*	*894*	
17. b) Final	**Reversal learning**	(Intercept)	1.721	1.500	1.949	989	0.001
		**Reward: seeds (vs worms)**	**-0.138**	**-0.234**	**-0.041**	**1007**	**0.006**
		**Shyness**	**-0.095**	**-0.153**	**-0.038**	**1013**	**0.002**
		Phylogeny	*0.029*	*0.000*	*0.070*	*912*	
		Capture site	*0.005*	*0.000*	*0.013*	*990*	
		Species	*0.009*	*0.000*	*0.028*	*864*	
18. a) Full	**Reversal learning**	(Intercept)	2.078	0.630	3.525	1003	0.005
		Food innovation	0.068	-0.073	0.207	1018	0.314
		Body condition	-0.139	-0.431	0.158	998	0.343
		Captive status: wild (vs dom)	-0.043	-0.344	0.258	1017	0.773
		Dietary generalism	-0.058	-0.158	0.041	1002	0.234
		**Reward: seeds (vs worms)**	**-0.132**	**-0.233**	**-0.030**	**1014**	**0.012**
		Fasting time	-0.017	-1.289	1.255	998	0.969
		Neophobia	0.014	-0.049	0.078	1004	0.664
		**Shyness**	**-0.090**	**-0.149**	**-0.031**	**1033**	**0.003**
		Phylogeny	*0.027*	*0.000*	*0.073*	*913*	
		Capture site	*0.005*	*0.000*	*0.015*	*974*	
		Species	*0.010*	*0.000*	*0.032*	*906*	
18. b) Final	**Reversal learning**	(Intercept)	1.721	1.500	1.949	989	0.001
		**Reward: seeds (vs worms)**	**-0.138**	**-0.234**	**-0.041**	**1007**	**0.006**
		**Shyness**	**-0.095**	**-0.153**	**-0.038**	**1013**	**0.002**
		Phylogeny	*0.029*	*0.000*	*0.070*	*912*	
		Capture site	*0.005*	*0.000*	*0.013*	*990*	
		Species	*0.009*	*0.000*	*0.028*	*864*	
19. a) Full	**Reversal learning**	(Intercept)	1.673	-0.006	3.366	1011	0.054
		Absolute brain size	-0.253	-1.012	0.516	1020	0.509
		Body condition	0.011	-0.478	0.504	1004	0.963
		Captive status: wild (vs dom)	-0.018	-0.327	0.292	1012	0.903
		Dietary generalism	-0.030	-0.123	0.063	1013	0.510
		**Reward: seeds (vs worms)**	**-0.146**	**-0.248**	**-0.045**	**1014**	**0.006**
		Fasting time	0.080	-1.204	1.359	1013	0.903
		Neophobia	0.007	-0.056	0.069	1011	0.822

		**Shyness**	**-0.090**	**-0.148**	**-0.031**	**1007**	**0.004**
		Phylogeny	*0.028*	*0.000*	*0.075*	*917*	
		Capture site	*0.005*	*0.000*	*0.015*	*996*	
		Species	*0.010*	*0.000*	*0.033*	*906*	
19. b) Final	**Reversal learning**	(Intercept)	1.721	1.500	1.949	989	0.001
		**Reward: seeds (vs worms)**	**-0.138**	**-0.234**	**-0.041**	**1007**	**0.006**
		**Shyness**	**-0.095**	**-0.153**	**-0.038**	**1013**	**0.002**
		Phylogeny	*0.029*	*0.000*	*0.070*	*912*	
		Capture site	*0.005*	*0.000*	*0.013*	*990*	
		Species	*0.009*	*0.000*	*0.028*	*864*	
20. a) Full	**Reversal learning**	(Intercept)	1.905	0.424	3.362	1010	0.013
		Relative brain size	-0.094	-0.622	0.432	997	0.705
		Body condition	-0.106	-0.411	0.212	1014	0.489
		Captive status: wild (vs dom)	-0.024	-0.334	0.295	1004	0.863
		Dietary generalism	-0.033	-0.130	0.062	1014	0.466
		**Reward: seeds (vs worms)**	**-0.144**	**-0.246**	**-0.043**	**1012**	**0.007**
		Fasting time	0.050	-1.224	1.323	1022	0.936
		Neophobia	0.009	-0.054	0.071	1006	0.785
		**Shyness**	**-0.090**	**-0.149**	**-0.031**	**1015**	**0.003**
		Phylogeny	*0.031*	*0.000*	*0.081*	*891*	
		Capture site	*0.005*	*0.000*	*0.015*	*983*	
		Species	*0.011*	*0.000*	*0.036*	*885*	
20. b) Final	**Reversal learning**	(Intercept)	1.721	1.500	1.949	989	0.001
		**Reward: seeds (vs worms)**	**-0.138**	**-0.234**	**-0.041**	**1007**	**0.006**
		**Shyness**	**-0.095**	**-0.153**	**-0.038**	**1013**	**0.002**
		Phylogeny	*0.029*	*0.000*	*0.070*	*912*	
		Capture site	*0.005*	*0.000*	*0.013*	*990*	
		Species	*0.009*	*0.000*	*0.028*	*864*	
21. a) Full	**Self-control**	(Intercept)	0.977	-0.495	2.455	1007	0.199
		Technical innovation	-0.039	-0.191	0.113	1013	0.584
		Body condition	0.265	-0.055	0.574	1016	0.106
		Captive status: wild (vs dom)	0.120	-0.155	0.396	990	0.371
		Dietary generalism	-0.027	-0.120	0.067	1011	0.546
		Reward: seeds (vs worms)	-0.081	-0.181	0.019	1026	0.118
		Fasting time	-0.048	-1.330	1.243	1015	0.941
		Neophobia	-0.011	-0.074	0.052	1005	0.736
		**Shyness**	**-0.106**	**-0.167**	**-0.046**	**1015**	**0.001**
		Phylogeny	*0.024*	*0.000*	*0.069*	*893*	
		Capture site	*0.002*	*0.000*	*0.006*	*982*	
		Species	*0.012*	*0.000*	*0.034*	*940*	
21. b) Final	**Self-control**	(Intercept)	1.317	1.085	1.563	1002	0.001
		**Shyness**	**-0.102**	**-0.161**	**-0.043**	**1023**	**0.002**
		Phylogeny	*0.033*	*0.000*	*0.084*	*918*	
		Capture site	*0.002*	*0.000*	*0.006*	*981*	
		Species	*0.012*	*0.000*	*0.035*	*884*	
22. a) Full	**Self-control**	(Intercept)	1.111	-0.336	2.560	1003	0.134

		Food innovation	0.026	-0.116	0.166	1020	0.697
		Body condition	0.229	-0.080	0.528	1014	0.146
		Captive status: wild (vs dom)	0.114	-0.164	0.394	1014	0.401
		Dietary generalism	-0.041	-0.142	0.060	1013	0.391
		Reward: seeds (vs worms)	-0.071	-0.174	0.031	1011	0.182
		Fasting time	-0.095	-1.394	1.175	1002	0.889
		Neophobia	-0.008	-0.072	0.056	1000	0.808
		**Shyness**	**-0.105**	**-0.165**	**-0.044**	**997**	**0.002**
		Phylogeny	*0.024*	*0.000*	*0.072*	*907*	
		Capture site	*0.002*	*0.000*	*0.006*	*979*	
		Species	*0.012*	*0.000*	*0.036*	*936*	
22. b) Final	**Self-control**	(Intercept)	1.317	1.085	1.563	1002	0.001
		**Shyness**	**-0.102**	**-0.161**	**-0.043**	**1023**	**0.002**
		Phylogeny	*0.033*	*0.000*	*0.084*	*918*	
		Capture site	*0.002*	*0.000*	*0.006*	*981*	
		Species	*0.012*	*0.000*	*0.035*	*884*	
23. a) Full	**Self-control**	(Intercept)	0.863	-0.827	2.536	1015	0.318
		Absolute brain size	-0.176	-0.933	0.605	1011	0.629
		Body condition	0.340	-0.181	0.840	1011	0.202
		Captive status: wild (vs dom)	0.127	-0.144	0.398	985	0.339
		Dietary generalism	-0.029	-0.119	0.061	997	0.502
		Reward: seeds (vs worms)	-0.078	-0.177	0.021	1026	0.125
		Fasting time	-0.054	-1.336	1.228	1015	0.934
		Neophobia	-0.012	-0.075	0.051	1016	0.712
		**Shyness**	**-0.104**	**-0.164**	**-0.044**	**1020**	**0.002**
		Phylogeny	*0.021*	*0.000*	*0.064*	*893*	
		Capture site	*0.002*	*0.000*	*0.006*	*969*	
		Species	*0.011*	*0.000*	*0.032*	*961*	
23. b) Final	**Self-control**	(Intercept)	1.317	1.085	1.563	1002	0.001
		**Shyness**	**-0.102**	**-0.161**	**-0.043**	**1023**	**0.002**
		Phylogeny	*0.033*	*0.000*	*0.084*	*918*	
		Capture site	*0.002*	*0.000*	*0.006*	*981*	
		Species	*0.012*	*0.000*	*0.035*	*884*	
24. a) Full	**Self-control**	(Intercept)	0.874	-0.574	2.310	1018	0.238
		Relative brain size	-0.289	-0.752	0.178	1016	0.204
		Body condition	0.289	-0.007	0.577	1025	0.063
		Captive status: wild (vs dom)	0.145	-0.120	0.410	1008	0.268
		Dietary generalism	-0.023	-0.108	0.063	1020	0.563
		Reward: seeds (vs worms)	-0.083	-0.183	0.015	998	0.102
		Fasting time	-0.011	-1.295	1.264	1008	0.972
		Neophobia	-0.010	-0.073	0.053	1013	0.768
		**Shyness**	**-0.102**	**-0.162**	**-0.041**	**1034**	**0.002**
		Phylogeny	*0.018*	*0.000*	*0.056*	*911*	
		Capture site	*0.002*	*0.000*	*0.006*	*955*	
		Species	*0.011*	*0.000*	*0.030*	*976*	
24. b) Final	**Self-control**	(Intercept)	1.317	1.085	1.563	1002	0.001

**Shyness**	**-0.102**	**-0.161**	**-0.043**	**1023**	**0.002**
Phylogeny	*0.033*	*0.000*	*0.084*	*918*	
Capture site	*0.002*	*0.000*	*0.006*	*981*	
Species	*0.012*	*0.000*	*0.035*	*884*	

Supplementary Table 6. Female and male mean number of trials to succeed in each behavioural test, for the two species for which females were tested. *t* and *P* are calculated using two-sided Welch's unpaired t-tests and *P.adj* using Benjamini & Hochberg false discovery rate correction.

Blue jay

	Females (*n*=3)	Males (*n*=10)	*t*	*P*	*P.adj*
Problem-solving (trials)	4.9	6.7	2.1450	0.1273	0.5092
Associative Learning (trials)	17.7	18.1	0.0793	0.9423	0.9423
Reversal learning (trials)	27.0	32.0	0.6341	0.5536	0.9423
Self-control (trials)	16.7	15.2	0.2512	0.8190	0.9423

European starling

	Females (*n*=4)	Males (*n*=12)	*t*	*P*	*P.adj*
Problem-solving (trials)	3.9	4.0	0.0522	0.9606	0.9606
Associative Learning (trials)	14.0	21.4	1.3980	0.1931	0.3862
Reversal learning (trials)	24.3	33.2	1.6000	0.1388	0.3862
Self-control (trials)	16.3	15.4	0.1461	0.8907	0.9606

Supplementary Table 7. Tests of repeatability for shyness and neophobia measurements. Repeatability is calculated with the RptR package using logged individual measurements as the dependent variable, the measurement day (1 - 4) and species as fixed effects, the bird ID as a random effect, and the bird ID as the grouping variable. (n = 203 individuals; each personality trait was measured 4 times).

Personality trait	*n*	*R*	SE	2.5%	97.5%	*P*	*P (permut)*
Shyness	810	0.319	0.040	0.236	0.392	3.52E-20	0.0010
Neophobia	801	0.125	0.036	0.058	0.195	4.21E-04	0.0010
