# Supplementary material for: Problem-solving skills are predicted by technical innovations in the wild and brain size in passerines
Source: Nat Ecol Evol. 2024 Feb 22;8(4):806–16. doi: 10.1038/s41559-024-02342-7 (PMC11009111; doi:10.1038/s41559-024-02342-7)
Supplement: Supplementary file 2 — Reporting Summary [file 41559_2024_2342_MOESM2_ESM.pdf]

nature portfolio

Corresponding author(s): Jean-Nicolas Audet

Last updated by author(s): Jan 5, 2024

Reporting Summary

Nature Portfolio wishes to improve the reproducibility of the work that we publish. This form provides structure for consistency and transparency in reporting. For further information on Nature Portfolio policies, see our Editorial Policies and the Editorial Policy Checklist.

Statistics

For all statistical analyses, confirm that the following items are present in the figure legend, table legend, main text, or Methods section.

n/a	Confirmed	
☐	☒	The exact sample size (n) for each experimental group/condition, given as a discrete number and unit of measurement
☒	☐	A statement on whether measurements were taken from distinct samples or whether the same sample was measured repeatedly
☐	☒	The statistical test(s) used AND whether they are one- or two-sided<br>*Only common tests should be described solely by name; describe more complex techniques in the Methods section.*
☐	☒	A description of all covariates tested
☐	☒	A description of any assumptions or corrections, such as tests of normality and adjustment for multiple comparisons
☐	☒	A full description of the statistical parameters including central tendency (e.g. means) or other basic estimates (e.g. regression coefficient) AND variation (e.g. standard deviation) or associated estimates of uncertainty (e.g. confidence intervals)
☐	☒	For null hypothesis testing, the test statistic (e.g. F, t, r) with confidence intervals, effect sizes, degrees of freedom and P value noted<br>*Give P values as exact values whenever suitable.*
☐	☒	For Bayesian analysis, information on the choice of priors and Markov chain Monte Carlo settings
☒	☐	For hierarchical and complex designs, identification of the appropriate level for tests and full reporting of outcomes
☐	☒	Estimates of effect sizes (e.g. Cohen's d, Pearson's r), indicating how they were calculated

Our web collection on statistics for biologists contains articles on many of the points above.

Software and code

Policy information about availability of computer code

Data collection	The collected behavioural dataset is available at: https://zenodo.org/records/10206756. Innovation and brain size data was obtained from published databases.
Data analysis	All analyses have been conducted in R version 4.3.0. The functions and packages we used are stated in the methods.

For manuscripts utilizing custom algorithms or software that are central to the research but not yet described in published literature, software must be made available to editors and reviewers. We strongly encourage code deposition in a community repository (e.g. GitHub). See the Nature Portfolio guidelines for submitting code & software for further information.

Data

Policy information about availability of data

All manuscripts must include a data availability statement. This statement should provide the following information, where applicable:

- Accession codes, unique identifiers, or web links for publicly available datasets
- A description of any restrictions on data availability
- For clinical datasets or third party data, please ensure that the statement adheres to our policy

Dataset and R code are available at https://zenodo.org/records/10206756.

Human research participants

Policy information about studies involving human research participants and Sex and Gender in Research.

Reporting on sex and gender	*Use the terms sex (biological attribute) and gender (shaped by social and cultural circumstances) carefully in order to avoid confusing both terms. Indicate if findings apply to only one sex or gender; describe whether sex and gender were considered in study design whether sex and/or gender was determined based on self-reporting or assigned and methods used. Provide in the source data disaggregated sex and gender data where this information has been collected, and consent has been obtained for sharing of individual-level data; provide overall numbers in this Reporting Summary. Please state if this information has not been collected. Report sex- and gender-based analyses where performed, justify reasons for lack of sex- and gender-based analysis.*
Population characteristics	*Describe the covariate-relevant population characteristics of the human research participants (e.g. age, genotypic information, past and current diagnosis and treatment categories). If you filled out the behavioural & social sciences study design questions and have nothing to add here, write "See above."*
Recruitment	*Describe how participants were recruited. Outline any potential self-selection bias or other biases that may be present and how these are likely to impact results.*
Ethics oversight	*Identify the organization(s) that approved the study protocol.*

Note that full information on the approval of the study protocol must also be provided in the manuscript.

Field-specific reporting

Please select the one below that is the best fit for your research. If you are not sure, read the appropriate sections before making your selection.

☐ Life sciences ☐ Behavioural & social sciences ☒ Ecological, evolutionary & environmental sciences

For a reference copy of the document with all sections, see nature.com/documents/nr-reporting-summary-flat.pdf

Ecological, evolutionary & environmental sciences study design

All studies must disclose on these points even when the disclosure is negative.

Study description	This study tested relationships between avian behavioural data collected in the field and literature data on innovation and brain size.
Research sample	We collected behavioural data on 203 individuals of 13 wild and 2 domesticated avian species.
Sampling strategy	The species were chosen based on their abundance where the study was conducted (Rockefeller Field Research Center). We aimed at reaching a sample size of >= 12 individuals per species to account for individual variation in the behaviour we measured. We expected that 15 species would be sufficient to test for associations with species-specific data on innovation and brain size.
Data collection	The behavioural data were collected by JNA, by observing captive birds performing on our battery of behavioural tasks.
Timing and spatial scale	The field seasons occured from 2018 to 2020.
Data exclusions	No data were excluded from the analyses.
Reproducibility	The same behavioural protocol was used for all individual of each species (total: 203 birds).
Randomization	The birds were tested in order of their capture. The behavioural tasks were not randomised since the test order is expected to strongly influence the performance; therefore, it was kept identical for all tested birds.
Blinding	The behavioural data were only analysed at the end of the field seasons; therefore, the results were unknown throughout the testing period.

Did the study involve field work? ☒ Yes ☐ No

Field work, collection and transport

Field conditions	Birds were captured in any weather condition. When conditions were hostile (e.g., raining), mist nets were visited more often.

Location	Rockefeller Field Research Center (Millbrook, NY, USA, 41° 46′ 3.0″ N, 73° 45′ 2.5″ W)
Access & import/export	Field work was conducted in compliance with all local and national regulations. Permits were issued by Rockefeller University (IACUC permit # 17084), New York State Department of Environmental Conservation (Banding permit # 198, Scientific collection permit # 2284), United States Fish and Wildlife Service (Permit # MB-45822C) and United States Geological Survey (Permit # 24130).
Disturbance	We collected only the number of birds necessary to obtain a sufficient sample size to conduct our analyses. Non-target species were immediately released.

Reporting for specific materials, systems and methods

We require information from authors about some types of materials, experimental systems and methods used in many studies. Here, indicate whether each material, system or method listed is relevant to your study. If you are not sure if a list item applies to your research, read the appropriate section before selecting a response.

Materials & experimental systems

n/a	Involved in the study	
☒	☐	Antibodies
☒	☐	Eukaryotic cell lines
☒	☐	Palaeontology and archaeology
☐	☒	Animals and other organisms
☒	☐	Clinical data
☒	☐	Dual use research of concern

Methods

n/a	Involved in the study	
☒	☐	ChIP-seq
☒	☐	Flow cytometry
☒	☐	MRI-based neuroimaging

Animals and other research organisms

Policy information about studies involving animals; ARRIVE guidelines recommended for reporting animal research, and Sex and Gender in Research

Laboratory animals	We collected behavioural data on canaries (Serinus canaria) and zebra finches (Taeniopygia guttata). Zebra finches were obtained from our domestic colony and canaries were bought from a local breeder.
Wild animals	We captured and collected behavioural data on the following species: White-throated sparrow (Zonotrichia albicollis); Chipping sparrow (Spizella paserina); Brown-headed cowbird (Molothrus ater); American goldfinch (Spinus tristis); American robin (Turdus migratorius); European starling (Sturnus vulgaris); Gray catbird (Dumetella carolinensis); House wren (Troglodytes aedon); White-breasted nuthatch (Sitta carolinensis); Black-capped chickadee (Poecile atricapillus); Tufted titmouse (Baeolophus bicolor); Blue Jay (Cyanocitta cristata); and Eastern phoebe (Sayornis phoebe). Birds were captured using mist nets and were brought in behaviour cages immediately. Except for a few birds that were sacrificed for another study, birds were released after the behavioural tests at their initial capture site.
Reporting on sex	To minimise the sample size, we used only males since the sex can potentially affect behavioural measures. We added females of two species (blue jay and European starling) because reaching a sufficient sample size of only males for these species proved to be challenging. The effect of sex was assessed for these species.
Field-collected samples	Birds were housed in an aviary kept at 70 degrees F. Lighting period was adjusted daily to reflect the natural photoperiod to minimise the stress on wild animals.
Ethics oversight	All procedures were approved by Rockefeller University, New York State Department of Environmental Conservation, United States Fish and Wildlife Service and United States Geological Survey.

Note that full information on the approval of the study protocol must also be provided in the manuscript.
